# Two Dimensional Structural Analysis and Expression of a New *Staphylococcus aureus* Adhesin Based Fusion Protein 

**Published:** 2012

**Authors:** Jamshid Faghri, Delavar Shahbazzadeh, Kamran Pooshang Bagheri, Sharareh Moghim, Hajieh Ghasemian Safaei, Bahram Nasr Esfahani, Hossein Fazeli, Rahmatolah Yazdani, Hamid Mirmohammad Sadeghi

**Affiliations:** 1 *Department of Bacteriology and Virology, Faculty of Medicine , Isfahan University of Medical Sciences, Isfahan, Iran*; 2 *Biotechnology Research Centre, Pasteur Institute of Iran, Tehran, Iran*; 3 *Department of Biotechnology, Faculty of pharmacy, Isfahan University of Medical Sciences, Isfahan, Iran *

**Keywords:** Adhesion, Clumping factor A, Fibronectin binding protein A, Fusion protein, Staphylococcus aureus, Two dimensional structure

## Abstract

**Objective(s):**

*Staphylococcus aureus *is a foremost source of numerous nosocomial and community acquired infections. Antibiotic therapy for vancomycin resistant *S. aureus* (VRSA) can not promise the eradication of infections. Since adhesion is the major route of infections, adhesin based vaccine could suppress *S. aureus* infections. Fibronectin binding protein A (FnBPA) and clumping factor A (ClfA) are major responsible adhesions involved in *S. aureus* infections, so they could be candidate vaccine molecules against an extensive range of infections. This project intended to express a new fusion protein construct and analysis of biological activity regarding binding activity.

**Materials and Methods:**

pfnbA- ClfA construct was transformed to *Escherichia coli BL21 (DE3).* Transformant *E. coli *were grown in LB broth and induced with IPTG and cellular extracts were separated on SDS–PAGE. RT-PCR was performed to verify expression. Binding activity of fusion protein was studied using human gingival fibroblast (HGF) cell line. D1-D3 protein from unpublished study was used as control.

**Results:**

The expected fusion protein fragment showed by SDS-PAGE. RT-PCR verified the existence of mRNA relating to expressed fusion protein. Binding activity of *S. aureus *decreased after treatment of HGF cells with fusion protein.

**Conclusion:**

In total, binding activity of fusion protein was approximately two fold lesser than D1-D3 protein. It is supposed that the fusion protein could not be attached to its ligand easily and would be more accessible to antigen presenting cells and consequently protective antibodies will be produced. This project is pending for *in vivo* infection study in animal model.

## Introduction

From the past decades to now, *Staphylococcus aureus *has been the major cause of life-threatening community-acquired and nosocomial infections. *S. aureus *can adhere to many cells and invade many organs causing the broad spectrum of important Staphylococcal diseases which include septicemia, wound infections, endocarditis, septic arthritis, toxic-shock syndrome, scalded-skin syndrome, and food poisoning (1). 

Before the advent of antibiotic, infections with *S. aureus *caused numerous deaths. *S. aureus *bacteremia resulted in a mortality rate of 80% (2). The introduction of penicillin in the 1940s considerably changed this situation. Quickly *S. aureus *resistant to penicillin appeared. Methicillin was first introduced in 1959 as a first generation of semisynthetic penicillins for the treatment of infections caused by penicillin-resistant SA. Just 2 years following its introduction, the first methicillin-resistant *S. aureus *(MRSA) was described (3) and the first nosocomial MRSA epidemic was reported soon afterwards (4). Numerous nosocomial MRSA outbreaks have occurred in most developed and developing countries from many geographical regions (5, 6). 

For many years vancomycin has been considered the first option among other drugs for the treatment of MRSA. However, 12 years earlier, Hiramatsu *et al* reported the first strain of *S. aureus *with reduced susceptibility to vancomycin (7) followed by 2 additional cases from the USA (8, 9). These earlier isolates were termed Vancomycin Intermediate *S. aureus *(VISA). However, in July 2002, things changed when the Centers for Disease Control (CDC) published the first documented report of *S. aureus *that was resistant to vancomycin as well as being resistant to methicillin (10, 11). The infection occurred in a diabetic patient with chronic renal failure who was undergoing peritoneal dialysis in a hospital in Michigan (12, 8). Additional cases of vancomycin resistance *S. aureus *(VRSA) have been reported from USA and other countries, to date (13-16). The potential clinical impact of these strains on the management of patients is very important. 

Since adhesin is the major route of *S. aureus* entrance to human body, immunization with adhesin molecules could protect the human against Staphylococcal diseases. Adhesin molecules in *S. aureus* belong to a family of surface proteins designated microbial surface components recognizing adhesive matrix molecules (MSCRAMM). *S. aureus* adhesins have efficient ability to promote adhesion to the extracellular matrix and cell associated receptors as well as protein ligands in plasma (17-19). These important characteristics made them significant targets for vaccination to suppress bacterial colonization and consequent possible infections (20). Among MSCRAMM family of microbial proteins, fibronectin binding protein (FnBP) and clumping factor are major adhesins which interfere with adhesin and invasion. The FnBP adhesins of *S. aureus *promote adhesion to tissue extracellular matrix (18, 19) indwelling medical devices, keratinocytes, endothelial cells, and traumatized tissues as well as internalization by different cell types (18, 21-25). 

Two tandem *fnb *genes, encoding FnBPA and FnBPB (18, 19), exist in *S. aureus* genome. FnBPA is present in all standard and clinical strains. Each of FnBPA and FnBPB possesses three consecutive 37- or 38-amino-acid D motifs; designated D1, D2, and D3 comprise a high-affinity fibronectin binding domain (26). Ligand-binding domain of the FnBPA protein has been used to induce adhesion-blocking antibodies (27, 28). D1, D1-D2, D2-D3, D1-D3, and similar synthetic peptides could not generate efficient blocking antibodies (29-31). The main reason is high binding affinity of these molecules to fibronectin that is broadly distributed in extracellular milieu, different cell surfaces and plasma. In such circumstances antigen binds to its ligand and antigen presenting cells can not efficiently phagocyte them thus antibody response is largely prohibited. 

The goal of this study is overcoming the problem via structural manipulation in amino acid sequences responsible for binding activity of fibronectin binding domain to prevent *S. aureus* infections. 

## Materials and Methods

***Bioinformatics design***

Conformational alteration in structure of fibronectin-binding domain especially in active motifs could lead to changes in biological activity. 

Binding domains in *fnb*A genes are highly conserved among *S. aureus* standard strains accordingly* S. aureus *subsp*. aureus* NCTC 8325 was selected as reference strain (ACCESSION NC_007795). 

The ability of binding to Fn is related to the C-terminal 20 amino acids of each D motif (32-34). Active binding motifs are the sequence GG (I/V)DF, alteration to either of the GG or IDF causes lack of binding to Fn (18, 19, 33). Mutational deletion in binding motifs are not recommended due to necessity of binding motifs in induction of antibody response. The other way to overcome this problem is conformational alterations in either binding motifs or binding domain via insertion mutation. For this purpose short peptides from binding domain of adhesins relating to S. aureus NCTC 8325 were selected as candidate insertion sequence. The candidate peptide shall be induced the mentioned alterations, preferably existed in all or near almost strains of *S. aureus*, and act as a good immunogen. 

Binding domain of *S. aureus* adhesins including elastin-binding protein (35), collagen binding protein (36), Bone sialoprotein binding protein (37), and laminin binding protein (38) were studied regarding these characteristics and finally C-terminal fragment of clumping factor A binding domain was selected as candidate insertion sequence.

ClfA is an important adhesin bind to fibrinogen and involved in colonization of implanted biomaterials or damaged endothelial surfaces at the site of endovascular infections (39). ClfA as a major virulence factor has a significant role in such infections (40-42). The Fibrinogen binding activity of ClfA has been localized to the N-terminal A region of this protein (43). Binding domain of ClfA is too large; thus a short sized fragment corresponding to C- terminal segment of ClfA binding domain was selected as candidate insertion sequence. It is proved that C- terminal segment of ClfA binding domain has efficient immunogenicity. This segment not only alters the 2-D conformation of FnBPA binding domain in silico but may also boost the immunogenicity of final fusion protein. 

***Bioinformatics*** ***analysis***

*Evaluation of homology*

The homology of amino acid sequence between fusion protein (derived from *S. aureus *NCTC 8325) and the other *S. aureus* strains was evaluated using BlastP. BlastP was performed to evaluate the homology between amino acid sequences of fusion protein and human proteins as well. 

*Prediction of 2-dimensional structure of FnBPA, FnBPA binding domain (D1-D3), ClfA, and C-terminal segment of ClfA binding domain using PSIPRED software in UCL Server*

FnBPA binding domain (D1-D3) and terminal segment of ClfA binding domain derived from their origin molecules FnBPA and ClfA and their possible structures were elucidated and compared with target molecules separately (44). 

*Prediction of 2-dimensional structure of the possible target molecules D1-D2-D3-ClfA, ClfA-D1-D2-D3, D1-D2-ClfA-D3, and D1-ClfA-D2-D3 using PSIPRED software (44)*

There are four possible combinations to construct the fusion protein that should be structurally predicted. Different combinations of D1, D2, D3, and terminal segment of ClfA binding domain were predicted and compared with one another. The best combination must possess the highest conformational alteration in total structure and in binding motifs. 

***Evaluation of restriction sites***

Restriction sites within FnBPA binding domain (D1-D3), terminal segment of ClfA binding domain, fusion protein, and *pET-15b* sequences were determined before using DNAMAN software. 

***The primers used to RT-PCR for fusion protein and D1-D3 designed by oligo analyzer software***

Candidate primers analyzed by oligo analyzer software regarding Tm, GC %, dG, 3'-tail GC, 3'-tail dG, molecular weight, self annealing, and loops. A 525 bp and a 348 bp RT-PCR product should be detected after electrophoresis to verification of mRNA concerning fusion of protein and D1-D3 respectively. 

***Selection of overhangs to overlapping the PCR products via hybridization***

Hybridization temperature of KVSGDL overhangs was evaluated. Amino acids KVS (Lys, Val, Ser) are located at the end of D1-D3. Amino acids GDL (Gly, Asp, Leu) are located at the beginning of terminal fragment of ClfA. The nucleotide sequences relating to KVS and GDL were incorporated in ClfA Fw primer and D1-D3 Rv primer respectively. In this manner, digested PCR products would be hybridized together via KVS (AAA GTA AGC) and GDL **(**GGT GAT TTA) overhangs. 

***Bacterial strains, genes, protein, peptide, plasmid vector, cell line, and primers***

*Escherichia coli *BL21 (DE3) (Cinnagen-Iran) was selected as expression host. *pfnbA-clfA* from the previous study (45) and *pD1-D3* (unpublished data) were used as expression vectors. Human gingival fibroblast (HGF1-PI 1) was used as cell line for adhesion assay. All of the primers used in this study were manufactured by TAG Copenhagen Company (Sweden). 

***Expression of fusion protein and D1-D3 construct***

The transformant E. coli DH5α was harbouring pfnbA-clfA from the previous study and pD1-D3 (unpublished data) cultivated in LB broth containing ampicillin. The plasmid vector extracted using alkali lysis method. The extracted vector transformed into E. coli BL21 (DE3) cells using standard CaCl_2_ method. The cells of this preculture were harvested, washed and grown at 37° C in Luria-Bertani broth containing 100 µg/ml ampicillin with constant shaking (120 RPM) to an A_600_ of 0.6 and induced at 37° C with 100 μM IPTG for 4 hr. Thirty min after induction 100 µg/ml rifampin was added to the suspension to inhibit protein synthesis in expression host. A separate culture for each construct was not induced and used as control. Induced and uninduced cells were harvested by centrifugation and the wet pellet resuspended in sonication buffer containing Tris, EDTA and 1 mg lysozyme/ wet pellet. The cell suspension disrupted in a laboratory sonicator (MSE) five times (30 sec pulse and 45 sec rest). Crude extract was filtered in 0.2 µm syringe filter and centrifuged at 13000 RPM to exclude the cell debris. 

***SDS-PAGE***

Protein concentration was measured at 280 nm in a UV photometer (Biometra- Germany). Crude extracts relating to *pfnbA-clfA*, *pD1-D3*, and uninduced cells were boiled for 5 min in sample buffer and separated on SDS–12.5% polyacrylamide gel at 110 volt for 3 hr in tris-glycine-SDS buffer (pH 8.3).

***RT-PCR for mRNAs transcribed from the ORF of fnbA-clfA***

For final verification of fusion protein expression, the mRNAs corresponding to *fnbA-clfA* ORF were extracted from the cell pellets using RNX solution (Cinnagen-Iran). The RT-PCR reactions should lead to production of 525 bp PCR products. cDNA synthesis was performed for induced and uninduced cells at 42 °C for 1 hr using 1 µl random hexamer (Fermentas), RT (200U/µl), 170 and 130 µg RNA for induced and uninduced cells respectively and reached to final volume of 20 µl with DEPC treated water.

cDNA synthesis followed by RT-PCR reaction using specific forward (5'-ATGGGCCAAAATAGCGGTAAC-3') and reverse primer (5'- CTCTGGAATTGGTTCAATTTC-3') against synthesized fnbA-clfA cDNA.

The RT-PCR mixture for fnbA-clfA ORF consisted of 3 µl cDNA**, **10 pm of forward and reverse primers, 1, 1.5, 3, and MgCl_2_, 200 µM of each dNTP, 1X PCR buffer, and 1 U Taq DNA polymerase (Cinnagen-Iran) and double-distilled water were added to achieve a final volume of 30 µl. A total number of 40 PCR cycles were run under the following conditions: DNA denaturation at 95 °C for 1 min (5 min for the first cycle), primer annealing at 50 °C for 1 min, and DNA extension at 72 °C for 2 min. PCR product was analyzed using a 1% ethidium bromide-stained agarose gel.

***RT-PCR for mRNAs transcribed from pD1-D3 ORF***

For final confirmation of D1-D3 expression, the mRNA corresponding to *D1-D3* ORFs was extracted from the cell pellets using RNX solution (Cinnagen-Iran). The RT-PCR reactions for D1-D3's mRNA should lead to production of 348 bp PCR products. cDNA synthesis is followed by RT-PCR reaction using specific forward (5'-ATGGGCCAAAATAGCGGTAAC-3') and reverse primer (5'-GCTTACTTTTGGAAGTGTATC-3') against synthesized D1-D3 cDNA.

cDNA synthesis was performed for induced and uninduced cells at 42 °C for 1 hr using 1µl random hexamer (Fermentas), RT (200 U/µl), 200 and 140 µg RNA for induced and uninduced cells respectively and reached final volume of 20 µl with DEPC treated water.

The RT-PCR mixture for fnbA-clfA ORF contained of 3 µl cDNA**, **10 pm of forward and reverse primers, 1.5, 3, and MgCl_2_, 200 µM of each dNTP, 1X PCR buffer, and 1 U Taq DNA polymerase (Cinnagen-Iran) and double-distilled water was added to achieve a final volume of 30 µl. PCR heating program was the same as for fnbA- ClfA.

***Adhesion assay***

A loopfull of bacteria subcultured in brain hear infusion (BHI) broth at 37 °C for 18-24 hr. 1.4 ml of heavy culture centrifuged at 7000 RPM for 5 min. The pellet were washed two times in TE buffer at 7000 RPM for 5 min and resuspended in 200 μl TE buffer. This concentrated suspension was used to prepare 0.5 McFarland standard by means of a spectrophotometer (SPECTRONIC 20D- Milton Roy). An OD equivalent to 0.1 at 625 nm was agreed as 0.5 McFarland standard. At this point the bacterial concentration is equal to 1.5×10^8^/ml. Fibroblasts (3×10^3)^ were cultured on coverslip in 1 ml RPMI 1640 with %10 FBS in a 24 well plate for 3 days in a humidified 37 °C, 5% Co_2_ incubator (Memmert). The wells and coverslips were washed with TE buffer two times and refilled with 1 ml TE buffer. Three descending concentrations of fusion protein and D1-D3 were prepared in TE buffer and 500 μl from each concentration were added to the wells and incubated at 37 °C for 1 hr. Two duplicate wells were used as growth control and adhesion control and no protein were added. The coverslips were washed with TE buffer two times. 100 μl bacteria equal to 1.5×10^7^ bacteria was added to wells, mixed, and incubated at 37 °C for 30 min. The coverslips were washed with 1ml TE buffer two times. The cells were fixed with 100 μl methanol, washed again with TE buffer, and stained with 50 μl crystal violet (0.02%) for 3 min. The coverslips were washed with 1 ml TE buffer three times, dried out at RT, and counted under microscope in 100 fields at 40X magnification and the average count calculated as result.

## Results

***BlastP***

The results demonstrated that the amino acid sequence of fusion protein is very similar to all published sequences (identity: 85-100%) corresponding to *S. aureus* Fibronectin-binding proteins A and B as well as Clumping factor A. No significant similarity was found between amino acid sequences of fusion protein and human proteins.

**Figure1:**
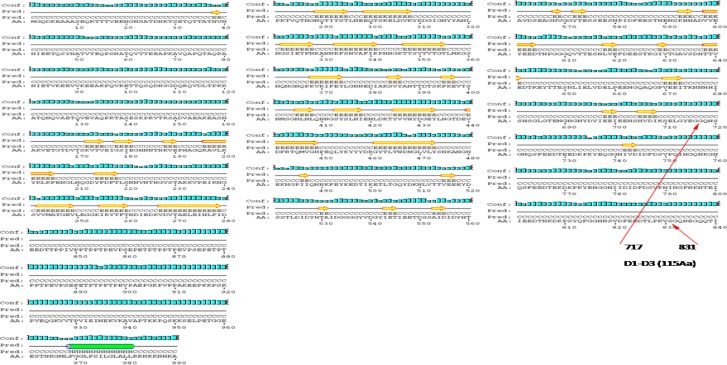
Prediction of 2-Dimensional structure of the FnBPA

**Figure 2:**
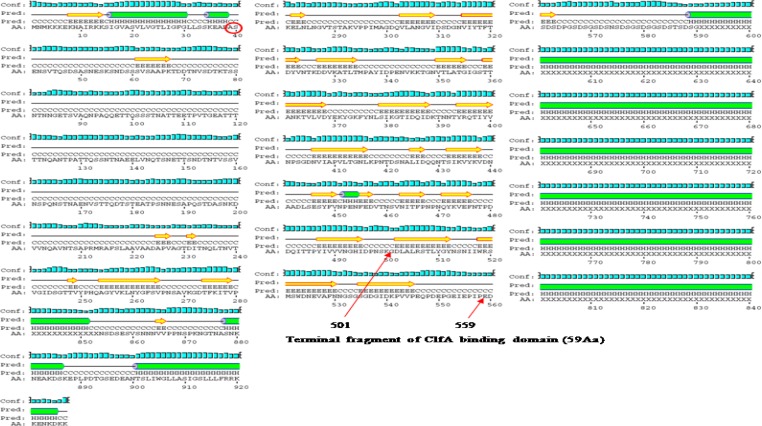
Prediction of 2-Dimensional structure of the ClfA

***Prediction of 2-dimensional structure of the FnBPA, ClfA, FnBPA binding domain (D1-D3), and terminal segment of ClfA binding domain***

2-D prediction was performed using PSIPRED software in UCL Server (44). Summarized results regarding total structure and binding motifs are listed in Table 2. Predicted conformation of FnBPA includes 38 beta strands, 40 coils, and 1 alpha helix. Binding domain is flanked by amino acid sequence number 717-831. 2-D structure of this domain within molecule consists of 1 beta strand and 2 coils (Figure 1).

Predicted conformation of ClfA includes 28 beta strands, 34 coils, and 6 alpha helixes. ClfA binding domain is located in C-terminal between amino acids 40 and 559.

Terminal fragment of ClfA binding domain (59 amino acid) is located between amino acids 501 and 559. 2-D structure of this fragment within molecule consists of 3 beta strands and 4 coils (Figure1). 

**Figure 3:**
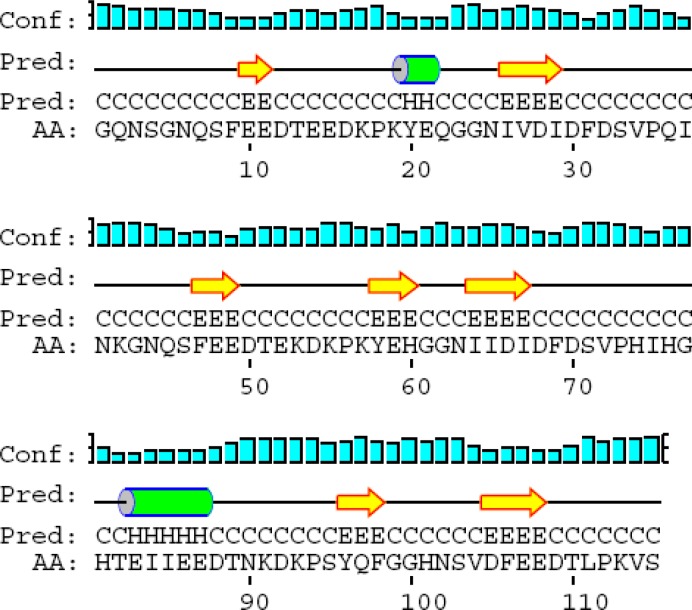
2-D structure of D1-D3

FnBPA binding domain (D1-D3) consists of 115 Amino acids flanked by amino acid sequence number 717-831. Its predicted conformation includes 7 beta strands, 2 alpha helixes, 10 coils. D1, D2, and D3 consist of 38, 38, and 39 amino acids respectively. Binding motifs in D1, D2, and D3 are “IDF”, “IDF”, and “VDF” respectively. Within IDF (number 29-31) in D1, “I” has beta strand and “DF” have coil structures. Within “IDF” (number 67-69) in D2, “I” has beta strand and “DF” have coil structures. Within “VDF” (number 104-106) in D3, “V” has coil and “DF” have beta strand structures. Its predicted conformation includes 7 beta strands, 10 coils, and 2 alpha helixes (Figure 3). 

ClfA binding domain consists of 59 amino acids and flanked by amino acid sequence number 501-559. Its predicted conformation includes 3 beta strands, 4 coils, and no alpha helix (Figure 1).

***Prediction of 2-dimensional structure of the possible recombinant molecules rD1-D2-D3-ClfA, rClfA-D1-D2-D3, rD1-D2-ClfA-D3, and rD1-ClfA-D2-D3 using PSIPRED software (*** 44 ***)***

Among four possible combinations to construction of fusion protein, rD1-D2-D3-ClfA had the highest conformational alteration in total structure and in binding motifs and was selected as candidate molecule. All of downstream bioinformatic analyses were performed based on this arrangement. 

Summarized results regarding total structure and binding motifs are listed in Table 1. Predicted conformation of rD1-D2-D3-ClfA includes 11 beta strands, 12 coils, and no alpha helix (Figure 5).

The detail results regarding 2D prediction of D1-D3 binding domain within FnBPA, terminal fragment of ClfA within ClfA, D1-D3 binding domain, terminal fragment of ClfA, rD1-D2-D3-ClfA, rClfA-D1-D2-D3, rD1-D2-ClfA-D3, and rD1-ClfA-D2-D3 outlined in Table 1.

**Figure 4:**
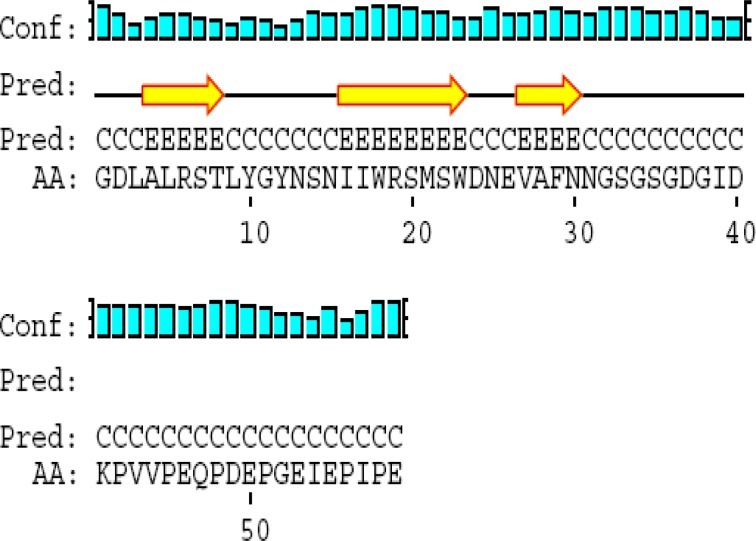
2-D structure of terminal segment of ClfA binding domain

**Figure 5:**
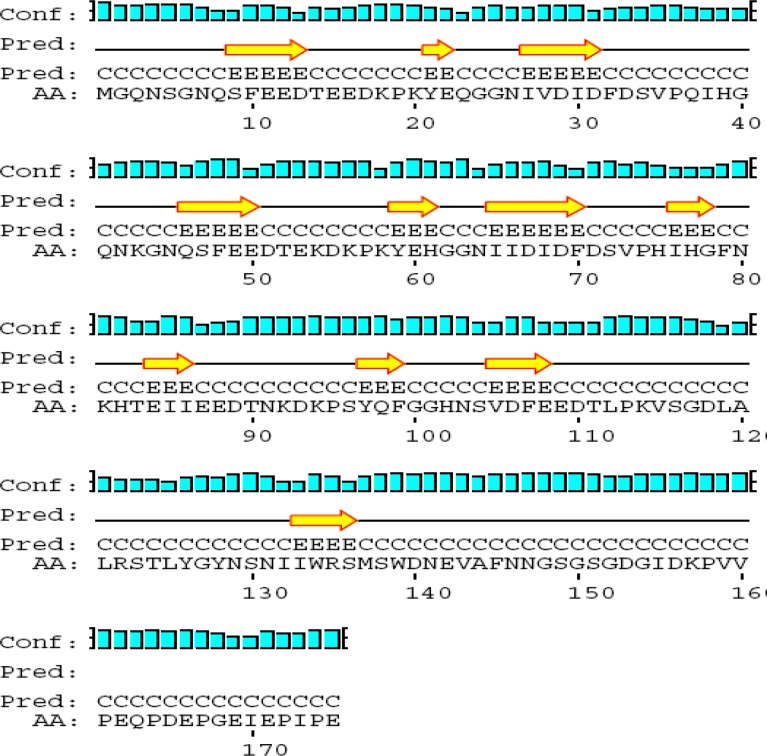
2-D structure of recombinant D1-D2-D3-ClfA

***SDS-PAGE of crude extracts relating to induced and uninduced cells for fusion protein***

**Figure 6:**
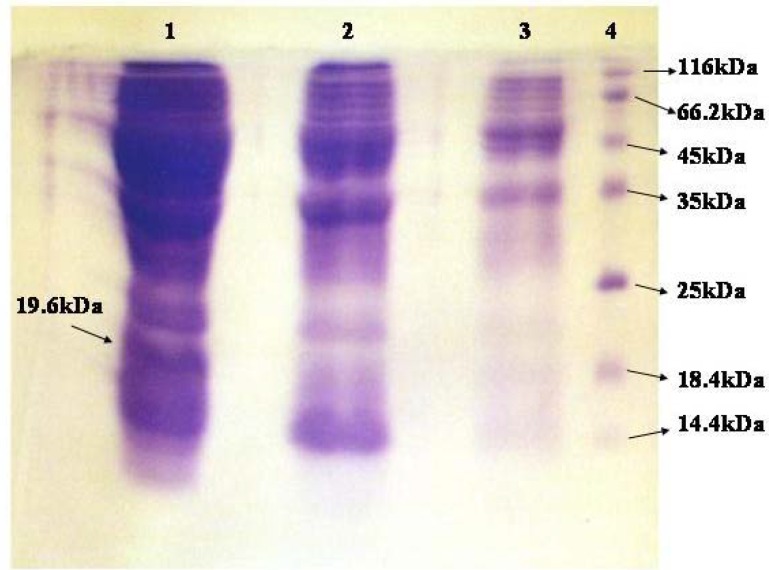
SDS-PAGE of crude extracts relating to induced and uninduced cells for fusion protein.

SDS-PAGE showed the expected fusion protein fragment in induced cells (Figure 6). Comparison of induced and uninduced cells showed a 19.6 kDa fragment in the crude extract of induced cells. Concentrations of crude extracts for induced and uninduced cells were 28.35 and 22.38 mg/ml respectively.

***SDS-PAGE of crude extracts relating to induced and uninduced cells for D1-D3***

**Figure 7:**
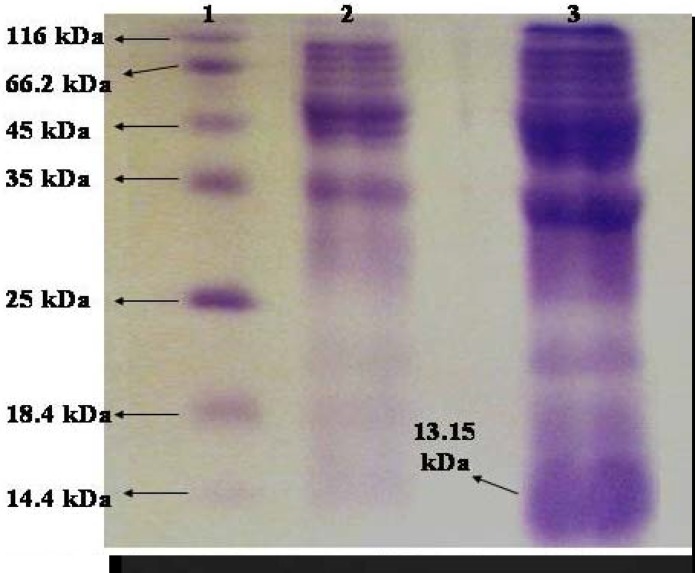
SDS-PAGE of crude extracts relating to induced and uninduced cells for D1-D3

Comparison of induced and uninduced bands showed a 13.1 kDa fragment in the crude extract of induced cells (Figure 7). Concentrations of crude extracts regarding the induced and uninduced cells, were 20.2 and 10 mg/ml respectively.

**Table1:** Detailed 2-D structure of adhesions, recombinant proteins and binding motifs

Structures Molecules	alpha helixes	beta strands	coils	IDF			IDF			VDF		
				I	D	F	I	D	F	V	D	F
D1-D3 binding domain within FnBPA	0	1	2	C			C			C		
Terminal fragment of ClfA within ClfA	0	3	4	____			____			____		
D1-D3 binding domain	2	7	10	B	C	C	B	C	C	C	B	B
Terminal fragment of ClfA	0	3	4	____			____			____		
rD1-D2-D3-ClfA	0	11	12	B	B	C	B	B	B	B	B	B
rClfA-D1-D2-D3	0	11	12	B	B	C	B	B	C	B	B	B
rD1-D2-ClfA-D3	2	9	12	B	C	C	B	B	C	C	C	B
rD1-ClfA-D2-D3	3	5	9	B	B	C	C	C	C	C	C	B

Lane 1. Crude extract relating to induced cells.

Lane 2. Crude extract relating to uninduced cells.

Lane 3. 1/10 diluted crude extract relating to uninduced cells.

Lane 4. Protein molecular weight marker (Fermentas). Concentration of each fragment is 0.1-0.2 mg/ml.

Lane 1. Protein Molecular Weight Marker (Fermentas). Concentration of each fragment is 0.1-0.2 mg/ml

Lane 2. Crude extract relating to Uninduced cells

Lane 3. Crude extract relating to Induced cells

***RT-PCR for fusion protein***

**Figure 8:**
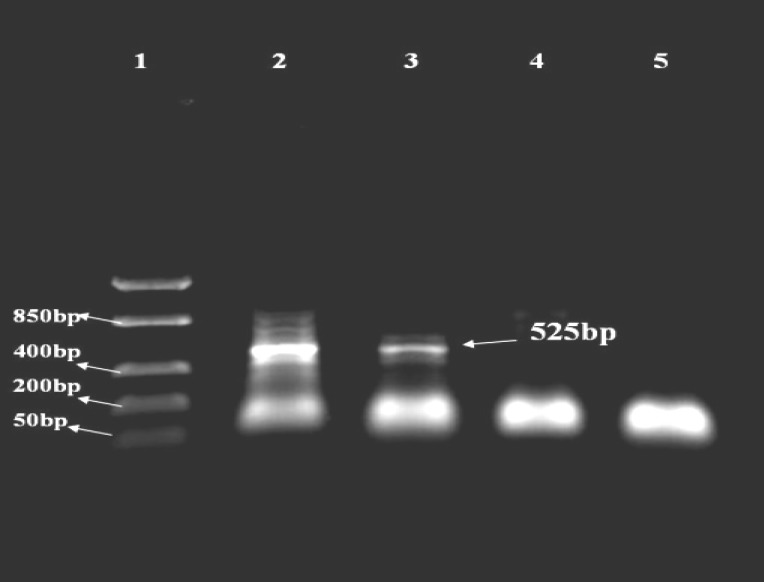
RT-PCR for mRNA related to fusion protein

**Figure 9:**
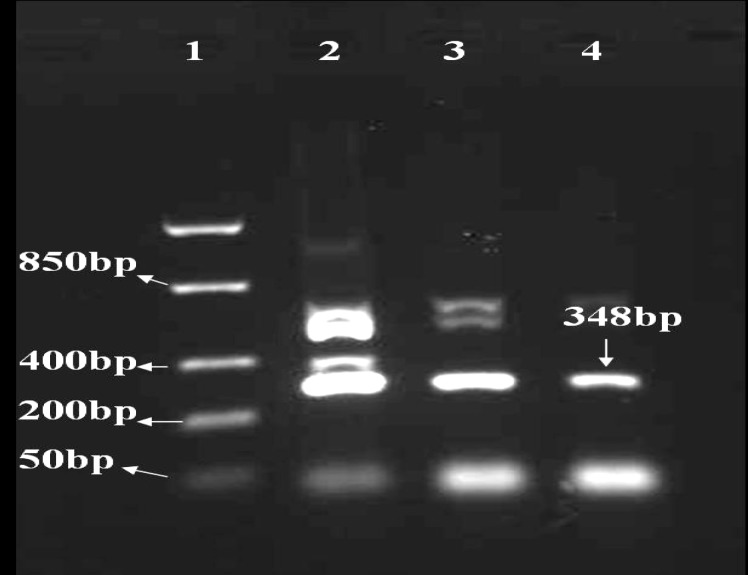
RT-PCR for mRNA related to D1-D3

The amplified fragments relating to mRNAs were transcribed from pfnbA-clfA and pD1-D3 constructs are shown in Figures 8 and 9. A 525 bp product was seen after electrophoresis in 1% agarose gel. Different concentrations of MgCl_2 _were applied in the test. The fragment amplified in 3 Mm MgCl_2_ was accepted as result. 

***RT-PCR for D1-D3***

A 348 bp product was observed in the following electrophoresis in 1% agarose gel. Different concentrations of MgCl_2 _were applied in the test. The fragment amplified in 1.5 Mm MgCl_2_ was accepted as result.

Lane 1. DNA ladder FastRuler, low range (Fermentas)

Lane 2-5. Ascending concentration of MgCl_2_: 5 Mm, 3 Mm, 1.5 Mm, 1Mm

Lane 1. DNA ladder FastRuler, low range (Fermentas)

Lane 2-4. Ascending concentration of MgCl_2_: 5 Mm, 3 Mm, 1.5Mm

Cell adhesion assay

**Figure 10:**
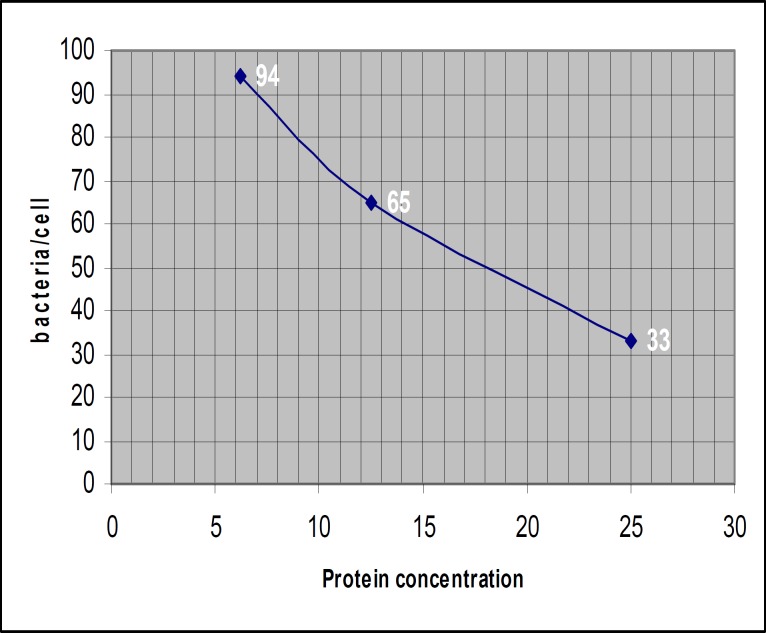
Average quantity of attached bacteria /cell in fusion protein group

**Figure 11:**
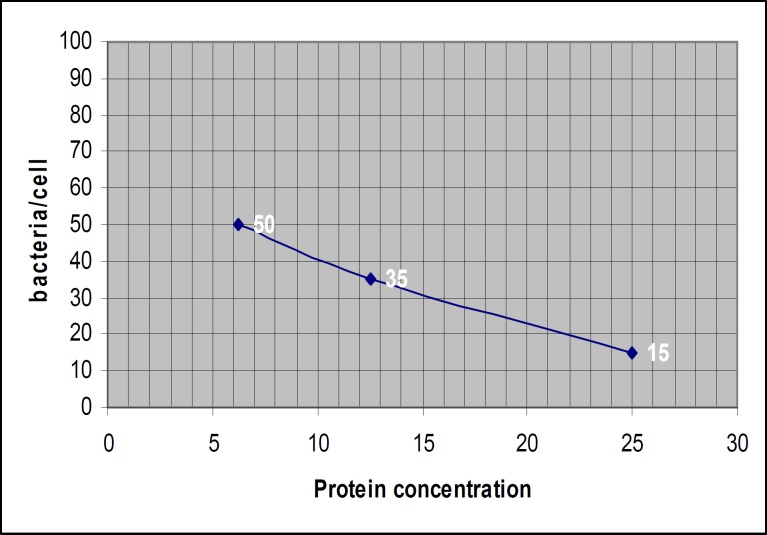
Average quantity of attached bacteria /cell in D1-D3 group

The attached bacteria on the surface of fibroblast in fusion protein and D1-D3 groups (induced and uninduced hosts) and quantity of attached bacteria/cell in three concentrations of fusion protein and D1-D3 (6.25, 12.5, and 25 mg/ml) are shown in Figures 10 and 11. The average quantity of attached bacteria for the crude extract regarding to uninduced host for fusion protein and D1-D3 vectors were approximately 110 bacteria/cell. The average quantity of attached bacteria for adhesion control group was about 115 bacteria/cell. 

Statistical analyses

Statistical analyses were performed using SPSS software (ver.12). 

Linear regression analysis was performed for fusion protein group and showed negative correlation (R= -0.987) between protein concentration and the number of attached bacteria. Linear regression analysis was performed in D1-D3 group and showed negative correlation (R= -0.994) between protein concentration and the number of attached bacteria. Comparing the results of attached bacteria between fusion protein group and D1-D3 group using FTEST confirmed that the variances are not significantly different (0.497). Student's t-Test showed that there is a borderline meaningful difference between the results in two groups (P= 0.05). 

## Discussion

Morbidity and mortality due to methicillin resistant *S. aureus* (MRSA) have been frequently reported from many geographical regions (46, 47). Intermediate or full resistance *S. aureus* strains to vancomycin have emerged during the past decade (48-51) and studies are being still made to search new alternative solutions. Annually, responsible health organizations in many countries routinely establish the annual significant budget to challenge staphylococcal diseases. One of the solutions to terminate this challenge would be inhibition of *S. aureus* entrance to body via adhesin and invasin molecules which could be attractive targets for vaccination strategies. 

The ascending concentrations of crude extract were 6.25, 12.5, and 25 mg/ml.

The efficacy of protection against *S. aureus* infections have been studied with the help of FnBPA or ClfA separately but the results could not meet the full requirements according to active immune protection (52, 53**-**55). Combination of protein antigens ClfA, FnBPA, and FnBPB appears to provide enhanced protection against prosthetic-device infection (56). It seems that combination of adhesin antigens may increase stimulation of the immune response system. The amino acid sequence relating to FnBPA and ClfA binding domain is highly conserved between *S. aureus* strains. Thus a fusion protein vaccine based on these two molecules could be applied against all of *S. aureus* infections.

*pfnbA-clfA* construct was designed to gather the characteristic of FnBPA and ClfA together (45). As the complete protein is not needed, binding domain of *fnbA* gene (D1-D3) and a C-terminal 59 amino acid from *clfA* binding domain were selected to construct a new fusion protein vector (55, 57- 60). *pD1-D3* vector was constructed for producing D1-D3 protein (unpublished data). This protein was used as positive control for bacterial adhesion. 

It is proposed that simultaneous production of antibodies against binding domains of FnBPA and ClfA may boost immunological response against forthcoming *S. aureus* infections.

Homology between amino acid sequence of *fnbA* gene and the other similar genes in the other *S. aureus* strains indicated that this kind of vaccine could be applied for the infections caused by other *S. aureus* strains. 

The data relating to prediction of 2-D structure of FnBPA, ClfA, fibronectin binding protein within FnBPA, terminal fragment of ClfA binding protein within ClfA, fibronectin binding protein, terminal fragment of ClfA binding protein and different combination of D1, D2, D3, and terminal fragment of ClfA were evaluated and compared with each other (Table1). Comparing the results showed that "D1-D3-ClfA fragment" had the most variations in binding motifs and total structure. Thus it was selected as the candidate molecule in this study.

RT-PCR results verified the existence of mRNA relating to fusion protein and D1-D3 peptide and hence the integrity of expression. Comparison of induced and uninduced bands in SDS-PAGE showed a 19.6 kDa and 13.1 kDa fragment in the crude extract of induced cells respectively. 

Binding activity of fusion protein measured microscopically in a cell culture model using human gingival fibroblast. The average quantity of adhered bacteria/cell was accepted as results. Increasing the concentration of fusion protein and D1-D3 led to decrease in quantity of attached bacteria (Figures 10, 11).

The results showed that the number of attached bacteria to fibroblast in the group, incubated with fusion protein were at least 2 fold higher than D1-D3 group. It means that adhesion property of fusion protein is 2 folds lower than D1-D3. In this condition, the fusion protein can not attach to its ligand easily and would be more accessible to antigen presenting cells. The consequence would be easier phagocytosis, presentation of target epitopes to lymphocytes, and production of protective antibodies. 

According to linear regression analysis in fusion protein group, increasing the protein concentration led to decrease in attached bacteria (R= -0.987). Based on linear regression analysis in D1-D3 group, increasing the protein concentration led to decrease in attached bacteria (R= -0.994). Comparing the results of attached bacteria between fusion protein group and D1-D3 group using student's t-test showed that there is a borderline difference in biological behavior of two molecules. It means that both of them have binding activity to fibroblast cells but with different activity (*P*= 0.05). Binding activity of fusion protein is approximately two folds lesser than D1-D3 protein. The average quantity of attached bacteria for fusion protein and D1-D3 in uninduced condition was the same and the results were equivalent to adhesion control group. 

Since the adhesion activity of fusion protein is lower than adhesion control group, it may be used as a candidate molecule in the next experiments. According to results, this fusion protein would be applied to prevention of VRSA infections as well as the other *S. aureus* infections.

Purification of fusion protein and D1-D3, adhesion assay with the other cell lines, administration of purified fusion protein to animal model and checking the efficacy of protection are depend on further evaluation.

## Conclusion

We studied the expression and biological activity of fusion protein. Binding activity of fusion protein was just about two fold lesser than D1-D3 protein. The result showed that the fusion protein might not be attached to its ligand easily and would be more reachable to antigen presenting cells and therefore protective antibodies will be produced. 

Purification of fusion protein and D1-D3, adhesion assay with the other cell lines, administration of purified fusion protein to animal model and checking the efficacy of protection are depend on further evaluation.
